# Umbilical cord medication in healthy full-term newborns: a before-after uncontrolled quality improvement study

**DOI:** 10.1007/s00431-020-03889-w

**Published:** 2020-12-07

**Authors:** Alessandra Coscia, Giovanni Boscarino, Maria Di Chiara, Francesca Faccioli, Roberto Pedicino, Elisa Onestà, Antonella Giancotti, Violante Di Donato, Benedetta Ronchi, Francesca Zantonelli, Alessia Russo, Chiara Mezzapiastra, Gianluca Terrin

**Affiliations:** 1 Neonatology Unit, Department of Public Health and Pediatrics, University of Turin, Turin, Italy; 2 Department of Maternal and Child Health Policlinico Umberto I, University La Sapienza, Rome, Italy

**Keywords:** Cord detachment, Cord stump, Newborns, Parents’ stress, Complications, *Arnica montana*, Omphalitis

## Abstract

Umbilical cord care can be a stressful practice for parents. Complications of cord care can increase neonatal morbidity and mortality. The extracts of *Arnica montana* (AM) have been reported to possess antibacterial, anti-inflammatory, antifungal, and immunomodulatory activities. We aim to demonstrate the efficacy of AM on cord detachment and parents’ stress level induced by cord medication in healthy full-term newborns. We enrolled full-term infants with a birth weight ≥ 2500 g in healthy conditions. Cord stumps of infants in the PRE-group were cleaned and dried, while cord stumps of infants in the POST-group were cleaned, dried, and medicated with a natural topic dermo-protective powder containing AM. After discharge, we interviewed parents on the stump status during follow-up visits in a pediatric office at 7 and 14 days of life, or by phone calls after follow-up visits. Long-rank test showed that time of cord separation of newborns in the PRE-group was significantly higher compared to that in the POST-group (*p* < 0.001). Parents of newborns in the PRE-group were significantly more stressed during cord medication compared to parents in the POST-group (2.0 (1.2 to 2.1) vs 1.0 (0.8 to 1.3), *p* = 0.011). Multivariate analysis showed a significantly linear relation with group assignment for cord separation (*p* < 0.001) and parents’ stress during the medication (*p* = 0.033).

*Conclusion*: The use of a natural topic dermo-protective powder containing AM reduces the time of cord separation, improves parents’ stress level, and reduces the risk of complications.

**Table:** 

**What is Known:** • *Cord stump care can be a stressful practice for parents.* • *Antiseptic treatment recommended for cord care could be associated with side effects such as burning and sensitization.*	
**What is New:** • *The medication of cord stump with a natural topic dermo-protective powder containing Arnica montana reduces time of cord detachment and of complication such as redness’, bleeding, or secretions.* • *The use of Arnica montana for cord medication may have a positive impact on the family, reducing parents’ stress, and the use of other medications.*

**Supplementary Information:**

The online version contains supplementary material available at 10.1007/s00431-020-03889-w.

## Introduction

Umbilical cord is an anatomic structure connecting the fetus and the placenta in the maternal uterus. Immediately after birth, the umbilical cord is usually cut. The cord stump dries and falls off within few days after birth. The stump can be an excellent medium for pathogenic bacteria [1]. Cord stump care can be considered a stressful practice for parents. The best practice for umbilical cord care remains controversial. Recent guidelines suggest cleaning and drying umbilical cord stump in babies born either in health facilities or at home, in low neonatal mortality settings [2]. In addition, they suggest the use of antiseptic treatments for infants born at home in high neonatal mortality setting and only for hindering application of harmful traditional substances for the umbilical stump [2]. The use of those medications may increase the time of cord detachment; it may be associated with substantial side effects and with increased risk of sensibilization to contact dermatitis and burning [3, 4]. Nevertheless, they are widely adopted despite no demonstrated benefits.

The extracts of *Arnica montana* (AM), a plant-derived natural product, have been reported to have antibacterial, anti-inflammatory, antifungal, and immunomodulatory activities [5]. Recent studies have demonstrated the efficacy and safety of AM for cord care, reducing the time of cord detachment and its complications [6, 7].

Starting from these considerations, we aimed to evaluate in a before-after uncontrolled quality improvement study the efficacy of a natural topic dermo-protective powder containing AM for cord care medication in reducing time of cord detachment. We also investigated parents’ stress level during umbilical cord medication, the occurrence of cord stump complications, the rate of use of other medications, parents’ perspective on medication difficulty, and behavior status of babies during cord stump medication.

## Materials and methods

We enrolled full-term infants with birth weight ≥ 2500 g, in healthy conditions, with Apgar score at 5 min ≥ 7, consecutively admitted in the postnatal ward of Policlinico Umberto I Hospital from 30 August 2019 to 23 April 2020. Newborns with congenital malformations, surgical conditions, and congenital infections and those that required hospitalization in the Neonatal Care Unit for post-natal complications or those with parents not speaking Italian or English language, were excluded. We obtained a written informed consent from all parents. Cord stumps of infants born from 30 August 2019 to 31 December 2019 (PRE-group) were cleaned and dried, while cord stumps of infants born from 1 January 2020 to 30 April 2020 (POST-group) were cleaned, dried, and medicated with a natural topic dermo-protective powder containing AM (Cicaben, Orsana® Itala S.r.l.).

We considered primary outcome the time of cord detachment. Secondary outcome was parents’ stress level during umbilical cord medication. We also evaluated the rate of cord stump complications, the rate of use of other medications, parents’ perspective on medication difficulty, and behavior status of babies during cord stump medication.

At the enrolment, we recorded data about infants (birth weight, gestational age, pH on cord blood, and Apgar at 5 min) and their mothers (age and parity). After discharge, we checked on the stump status through follow-up visits in a pediatric office within 2 weeks of life. At the time of cord detachment, parents filled in a questionnaire on their level of stress during medication and on the level of difficulty of medication. The questionnaire fulfilled required about 5 to 10 min. Parents were instructed to fulfill the questionnaire by researchers unaware of the study aims, at 7 and 14 days of life, during the follow-up visits. Parents of babies with persistence of cord stump at the 2nd visit received phone interviews within the next 2 weeks. The stress of parents during medication was evaluated by the administration of a specific questionnaire reporting a scale from 1 (very low) to 10 (very high). Level of difficulty of medication for the parents was evaluated by a questionnaire and self-reported in a scale from 1 (very easy) to 10 (very difficult). Behavior status of the babies during cord stump medication was classified as quiet, fussy, and crying, and it was reported by the parents in a specific data form. During the follow-up visits, a physician, blinded to group assignment, used to examine the cord stump for the possibility of complications (i.e., redness, bleeding, and secretion) and asked parents whether, before the cord detachment, they observed something wrong.

Statistical analysis was performed using the Statistical Package for Social Science software for Microsoft Windows (SPSS Inc. – IBM Corp ©, Chicago, IL), version 25.0. Time of cord detachment is described graphically using a Kaplan-Meier plot. The Kaplan-Meier method was used to estimate the probability of cord detachment at 15 days of life in each study group, and the resulting functions were compared with the log-rank test Mann-Whitney *U* test. The mean and standard deviation summarized continuous variables. We compared the two groups using the chi-square test for categorical variable and *t* test or Mann-Whitney for paired and unpaired variables.

We applied Cox regression analysis considering to be dependent variable our primary outcome and as covariates the variables resulted significantly different (*p* < 0.05) between the two study groups from univariate analysis and those suggested by the literature such as birth weight, Apgar score at 5 min, spring season (no or yes), type of delivery (vaginal delivery or cesarean section), and group assignment (PRE or POST) [8, 9]. In addition, we performed a second Cox regression analysis considering to be covariates the variables resulted marginally different (*p* < 0.2) between the two groups from univariate analysis (male sex and primiparity) and variables regarding parents’ status (primiparity, age of the mother, and difficulty of cord medication) that we hypothesize that could have influenced time of cord detachment.

We applied linear regression analysis considering to be dependent variable our secondary outcome (parents’ stress level) and as covariates those variables that could have influenced this outcome (age of the mother, difficulty of cord medication, primiparity) and group assignment (PRE or POST). The level of significance for all statistical tests was 2-sided (*p* < 0.05). On the basis of our preliminary data, collected at the same hospital where the study was performed, we calculated for primary outcome a minimum sample size of 156 patients (95% of power, 0.05 of type 1 error, 2-tailed test, dropout 30%) to demonstrate a difference of 2 days (8 vs 10 days, SD 3 days) in cord detachment. We increased at 230 patients the sample size, when we would demonstrate an increase of at least of 10% in the secondary outcome (90% of power, 0.05 of type 1 error, 2-tailed test, dropout 30%).

## Results

**Fig. 1:**
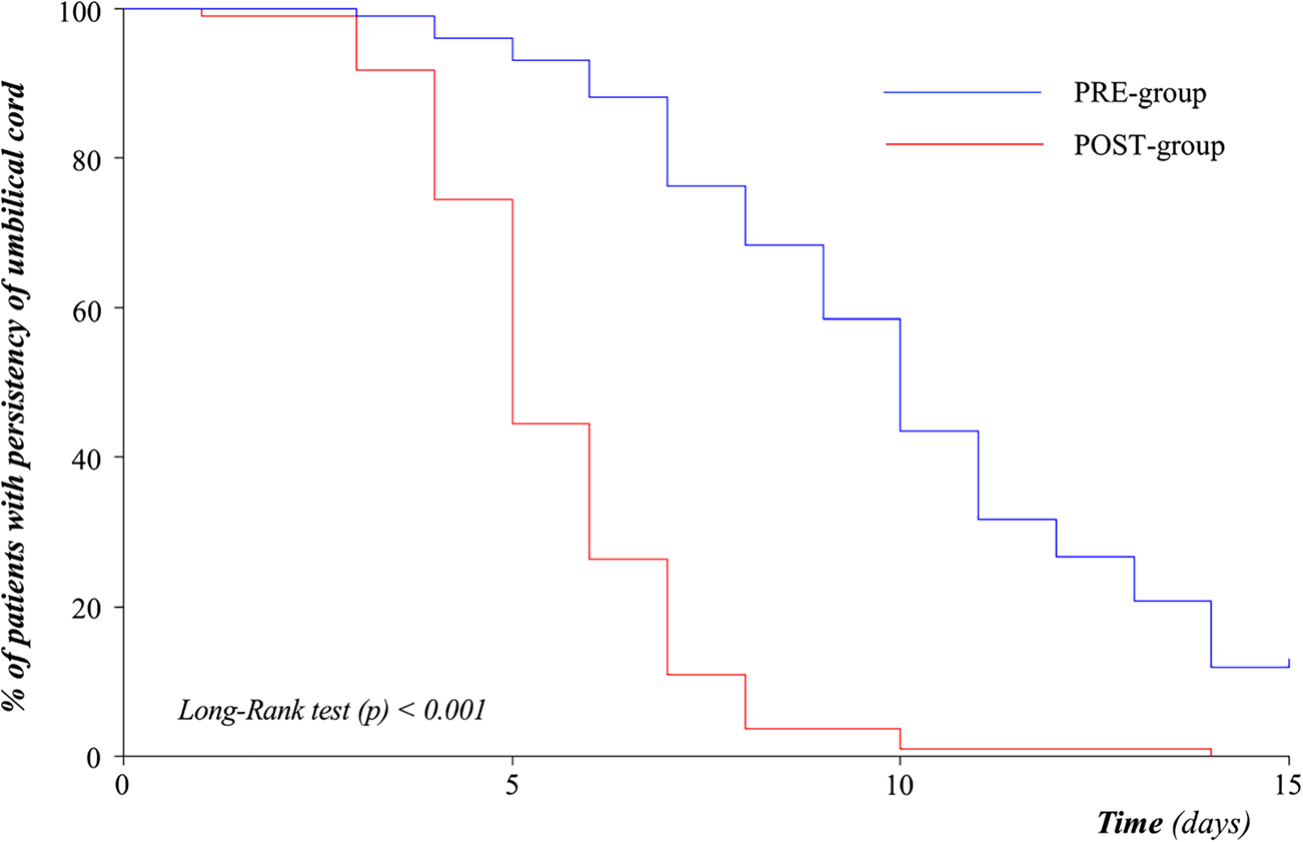
Duration of umbilical cord persistency

**Fig. 2:**
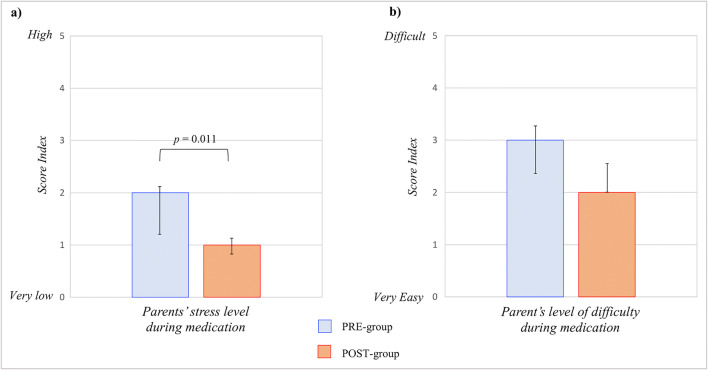
Umbilical cord medication: parents’ perspective

We considered eligible 239 newborns and we enrolled 230 subjects as shown in Supplementary Figure 1. The two groups were similar for baseline clinical characteristics (Table 1). The time of cord detachments was significantly higher in the PRE-group than in the POST-group (PRE 10.5 ± 4.1 days vs POST 5.6 ± 1.9 days, *p* < 0.001). The Kaplan-Meier functions showed a significant lower difference in the time of cord detachment of newborns in the POST-group (Fig. 1). Parents’ stress level during cord care of newborns in the PRE-group was significantly higher compared to that in parents in the POST-group, as shown in Fig. 2a. There was no significant difference in the level of difficulty for parents in the two study groups (Fig. 2b). The rate of complications was significantly lower in the POST-group compared with that in the PRE-group (Supplementary Figure 2). The complications that we observed were redness, bleedings, and purulent secretions (Supplementary Figure 2). In a sub-analysis, we separated all enrolled newborns in two sub-groups: (i) cord detachment within 5 days of life; (ii) cord detachment after 5 days of life. We found a significant higher rate of complications in newborns with a cord detachment occurring after 5 days of life ((i) 2.9% vs (ii) 23.9%, *p* < 0.001).

**Table 1:** Baseline characteristics of study population

	PRE-group (*n* = 100)	POST-group (*n* = 110)	*p*
Gestational age (weeks)	39 ± 1	39 ± 1	0.271
Birth weight (g)	3245 ± 373	3273 ± 422	0.607
Primipara mother, *N* (%)	57 (57.0)	76 (69.1)	0.085
Age of mother (years)	33 ± 5	32 ± 4	0.103
Cesarean section, *N* (%)	46 (46.0)	55 (50.0)	0.583
Male sex, *N* (%)	53 (53.0)	43 (39.1)	0.052
Apgar score at 5 min	10 ± 0	10 ± 1	0.221
pH on cord blood	7.3 ± 0.1	7.3 ± 0.1	0.500
Base excess on cord blood	− 5.7 ± 10.9	− 4.8 ± 2.9	0.389

Data were expressed as median ± standard deviation, when not specified

In addition, the percentage of parents using other medications was significantly higher in the PRE-group compared with that in the POST-group (29% vs 0%, *p* < 0.001). Behavior status of the babies during cord stump medication was similar in the two groups (Supplementary Figure 3).

Cox regression analysis showed a significant relation between the primary outcome and POST-group, spring season, and primiparity (Table 2 and Supplementary Table 1). Multivariate analysis showed that parents’ stress level depended on cord difficulty level and POST-group assignment (Table 2).

**Table 2:** Regression analysis to evaluate the influence of covariates on primary and secondary outcomes

		Std. Err.	*B*	*p* value	95% CI
Cord detachment timing (*χ*^2^ = 109.251, *p* value < 0.001)	Birth weight	0.000	0.000	0.384	0.999 to 1.000
	5-min Apgar score	0.132	0.126	0.340	0.875 to 1.470
	Spring season	0.196	− 0.590	*0.003*	0.378 to 0.814
	Type of delivery	0.145	− 0.032	0.825	0.729 to 1.287
	Group assignment	0.188	− 1.344	*< 0.001*	0.180 to 0.377
Parents’ stress level (*R*^2^ = 0.136, *R*^2^ adj. = 0.119)	Primiparity	0.258	− 0.022	0.931	− 0.531 to 0.487
	Age of mother	0.027	0.029	0.289	− 0.025 to 0.082
	Cord difficulty level	0.059	− 0.287	*< 0.001*	− 0.403 to − 0.171
	Group assignment	0.241	0.517	*0.033*	0.042 to 0.993

Entries in italic underline the statistical significance of the values

## Discussion

We demonstrated that the use of a natural topic dermo-protective powder containing AM reduces the time of cord detachment and its related complications. This kind of medication also decreases parents’ stress during umbilical cord medication within the first days of life. Application of a natural topic dermo-protective powder containing AM reduces the use of other medications, resulting in a cost saving for the family.

Two studies have evaluated the efficacy of *Arnica* on cord detachment and of other types of medications [6, 7]. Guala et al., in a controlled clinical trial, compared different six types of cord medication, focusing on the time of cord detachment [6]. The six groups included (1) clean of cord stump with sterile solution; (2) gauze dressing and 70% alcohol; (3) micronized pure colloid silver benzyl preparation; (4) micronized pure silver spray and a napkin folded below the stump; (5) micronized pure silver powder; and (6) AE. They found the longest time of detachment among infants in the control and alcohol groups and the shortest time among infants in the *Arnica* group. However, in this study, baseline clinical characteristics and the effects of confounding variables on primary outcome were not evaluated. Our results confirmed positive effects of AM medication, in a before-after uncontrolled quality improvement study, on time of cord detachment also when corrected for birth weight, Apgar score at 5 min, spring season, type of delivery, primiparity, age of the mother, and difficulty of cord medication. According with Shoaeib et al., we found a relation between time of cord detachment and the spring season [9]. In a large non-controlled study, Perrone et al. analyzed 6323 newborns treated with *Arnica* [7]. In their population, 89% of umbilical cords detached within 4 days of life. According with these results, in a before-after uncontrolled quality improvement study, we observed a significant earliest time of cord detachment in newborns medicated with AM.

Our findings demonstrated that the use of a natural topic dermo-protective powder containing AM may reduce parents’ stress related with cord stump medication. Previous studies found a better behavior status of parents using additional medications for the care of umbilical cord of their newborns [10, 11]. However, the use of medications such as chlorhexidine or alcohol 70% may increase the time of cord separation [1, 12]. Our findings suggest that medication of umbilical cord with a natural topic dermo-protective powder may contemporarily reduce parents’ stress and time of cord separation.

We confirmed the reduction of complications related to umbilical cord care associated with the use of a natural topic dermo-protective powder containing AM, observed in previous studies [6, 7]. Efficacy of products containing AM could be related to its antibacterial and anti-inflammatory activities, mainly attributed to the presence of flavonoids and phenolic compounds [5]. Flavonoids may promote anti-inflammatory mechanisms inhibiting reactive oxygen and nitrogen species [13]. These metabolites also switch off the activities of enzymes involved in free radical production and modulate different intracellular signaling pathways in immune cells [13]. The immunomodulatory activity of AM has been associated to polysaccharide fractions of AM flowers that increase the macrophage release of tumor necrosis factor, display an anti-complementary activity, and participate in promoting phagocytosis [14]. In addition, thymol derivatives, found in the roots of AM, have been reported to have antibacterial and antifungal activities, whereas the essential oil extracted from the roots shows antiphlogistic action [5].

However, it is not possible to exclude that the effects observed in our study could be due to the presence of other substances such as zinc oxide, cornstarch, arginine, allantoin, or magnesium oxide. Indeed, some of these substances have been recently associated to antibacterial activities [15, 16].

Finally, we found that parents of newborns in the PRE-group used other medications under either medical prescription or nurses’ and family’s advices. The most common medications were alcohol 70%, chlorhexidine, or other pharmaceutical products. In their review, Imdad et al. did not find advantages of application of either alcohol 70% or chlorhexidine in reducing time of detachment [1]. Furthermore, they did not find benefits of alcohol application in reducing colonization by pathogens [1]*.* Our results suggest that the use of a natural topic dermo-protective powder containing AM may reduce prescription of other unnecessary medications. However, further studies are needed to evaluate the effects of AM on bacterial colonization.

This study should be balanced with several study limitations. The association between AM and our primary and secondary outcomes may be related to the effects of chance (random error), bias, or confounding factors.

The two groups were similar for baseline clinical characteristics. We excluded newborns with congenital malformations and congenital infections or those requiring more than 7 days of hospitalization for post-natal complications, because they could have influenced time of cord detachment, parents’ stress level during medication, and all above the complications. Other confounding variables, unknown or not considered in our statistical analysis, may have influenced the study results.

This is not a randomized trial, but a before-after design remains the most practical method for studying an intervention [17]. However, as previously described, the use of historical control group could overestimate the benefit of a new treatment and this design could not control the use of other medications by parents [17]. To limit observer bias, the data for the analysis were collected by researchers not involved in eligibility assessment and who were unaware of group assignment. We discussed and defined a protocol for the collection, measurement, and interpretation of data before starting the study. Finally, a blinded statistician performed the data analysis.

We divided the two groups on a temporal basis. This has increased the risk of bias. Despite no changes in the policies care during the entire study period and similar baseline characteristics of the two groups, it is not possible to exclude that unknown differences in the clinical practice or changes in the medical staff composition may have influenced the results. In addition, stress is a not objective variables and this could influence the results according to parents’ personality. Our trial was not powered and focused to evaluate the incidence of omphalitis. It has been demonstrated that the rate of omphalitis depended on prenatal and perinatal practices, cord care strategies, and delivery venue (home vs hospital) [18]. In a developed country in good clinical condition, incidence is approximately 1 for 1000 newborns [18]. In addition, our score scales were not validated. To date, there are many validated scores to measure general stress level. However, these scores are not focused in measuring parents’ stress level specifically related to cord stump medication. In order to address this aim, we did not use a validated score, but we had preferred to administer to the parents’ singular questions that were analyzed and reported separately in the “Results” section of the study. Further, multicenter RCTs or controlled before-after studies are advocated to confirm our results.

The use of a natural topic dermo-protective powder containing AM may represent a further duty for the parents during their care of the babies in the first days of life and as stressful condition for the babies. We demonstrate that difficulty levels during cord care for parents using a natural topic dermo-protective powder containing AM and behavior of the babies were similar between the two study groups.

In conclusion, cord care medication with a natural topic dermo-protective powder containing AM is an efficacy strategy in a health care setting. Promising results observed in full-term newborns suggest possible use of AM also in preterm newborns.

## Supplementary Information

ESM 1 (PNG 53 kb)

High resolution (TIFF 83 kb)

ESM 2 (PNG 149 kb)

High resolution (TIFF 130 kb)

ESM 3 (PNG 102 kb)

High resolution (TIFF 124 kb)

ESM 4 (DOCX 16 kb)

## Data Availability

Data are available from the Department of Maternal and Child Health Policlinico Umberto I Hospital, La Sapienza University of Rome, Italy, Institutional Data Access for researchers who meet the criteria for access to confidential data.

## Abbreviations

AM: *Arnica montana*
