# Supplementary figures and images for: Umbilical cord medication in healthy full-term newborns: a before-after uncontrolled quality improvement study
Source: Eur J Pediatr. 2020 Dec 7;180(2):505–11. doi: 10.1007/s00431-020-03889-w (PMC7813727; doi:10.1007/s00431-020-03889-w)

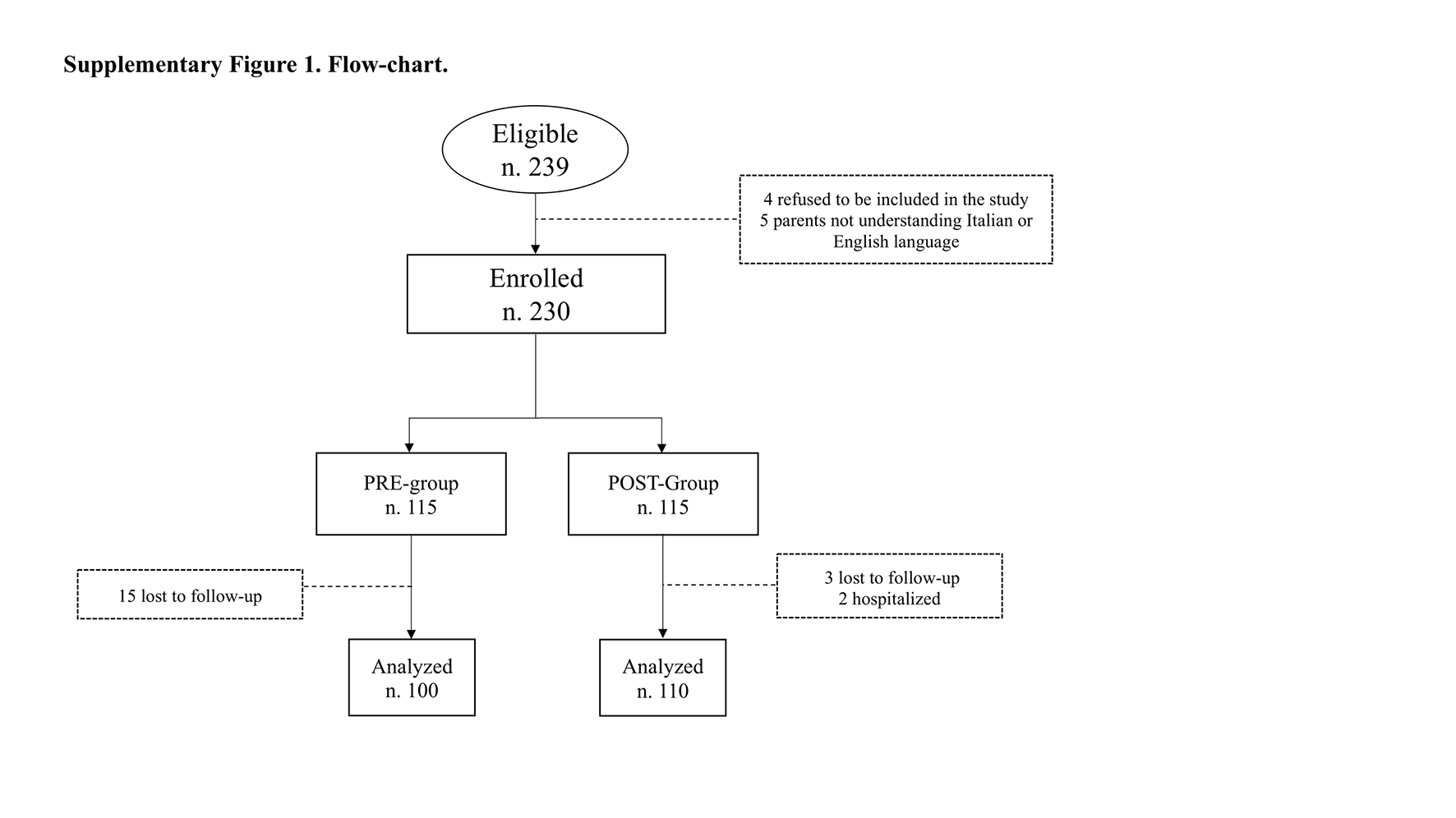

Supplement: Supplementary file 1 — (PNG 53 kb) [file 431_2020_3889_Fig3_ESM.png]

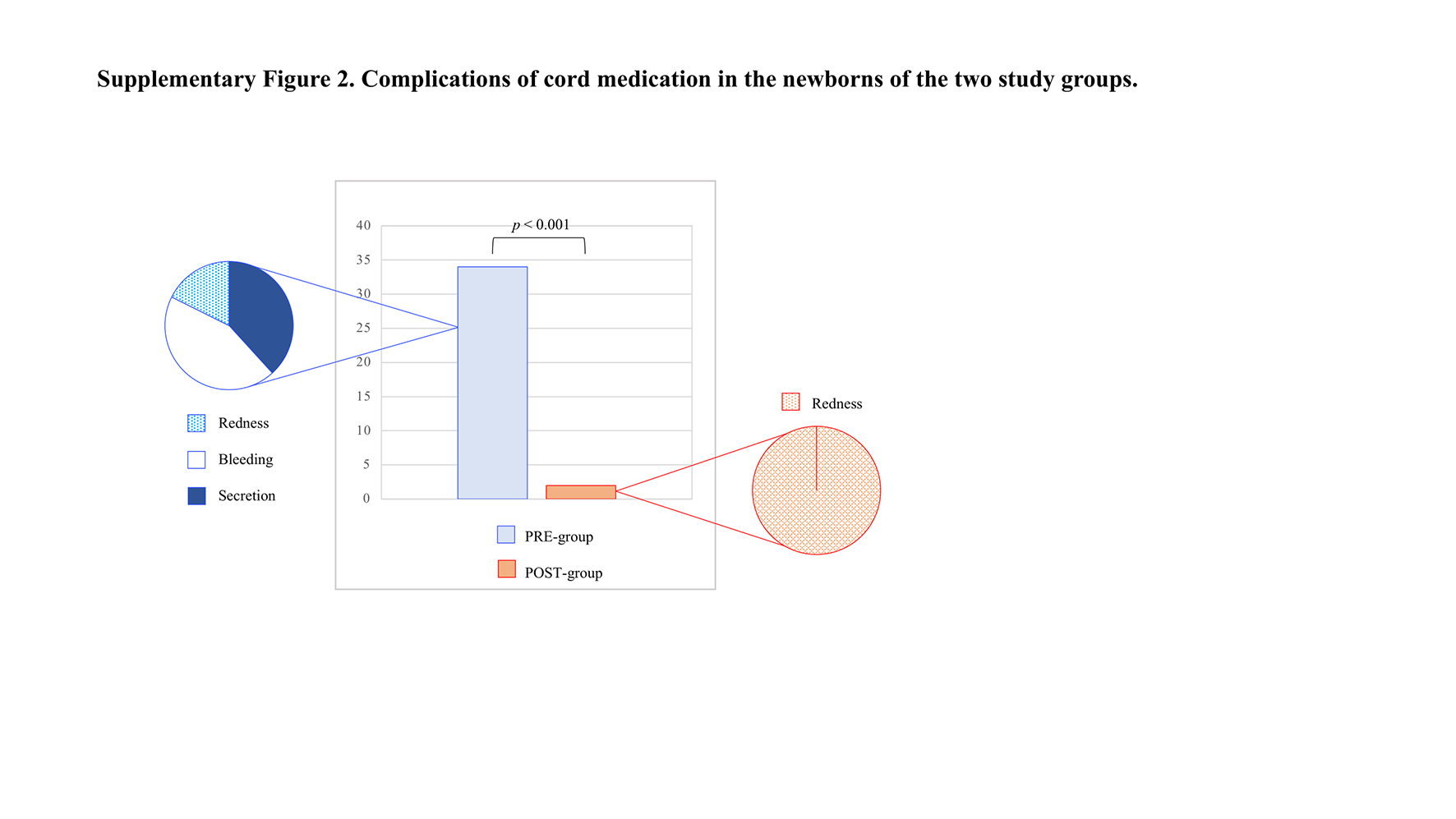

Supplement: Supplementary file 3 — (PNG 149 kb) [file 431_2020_3889_Fig4_ESM.png]

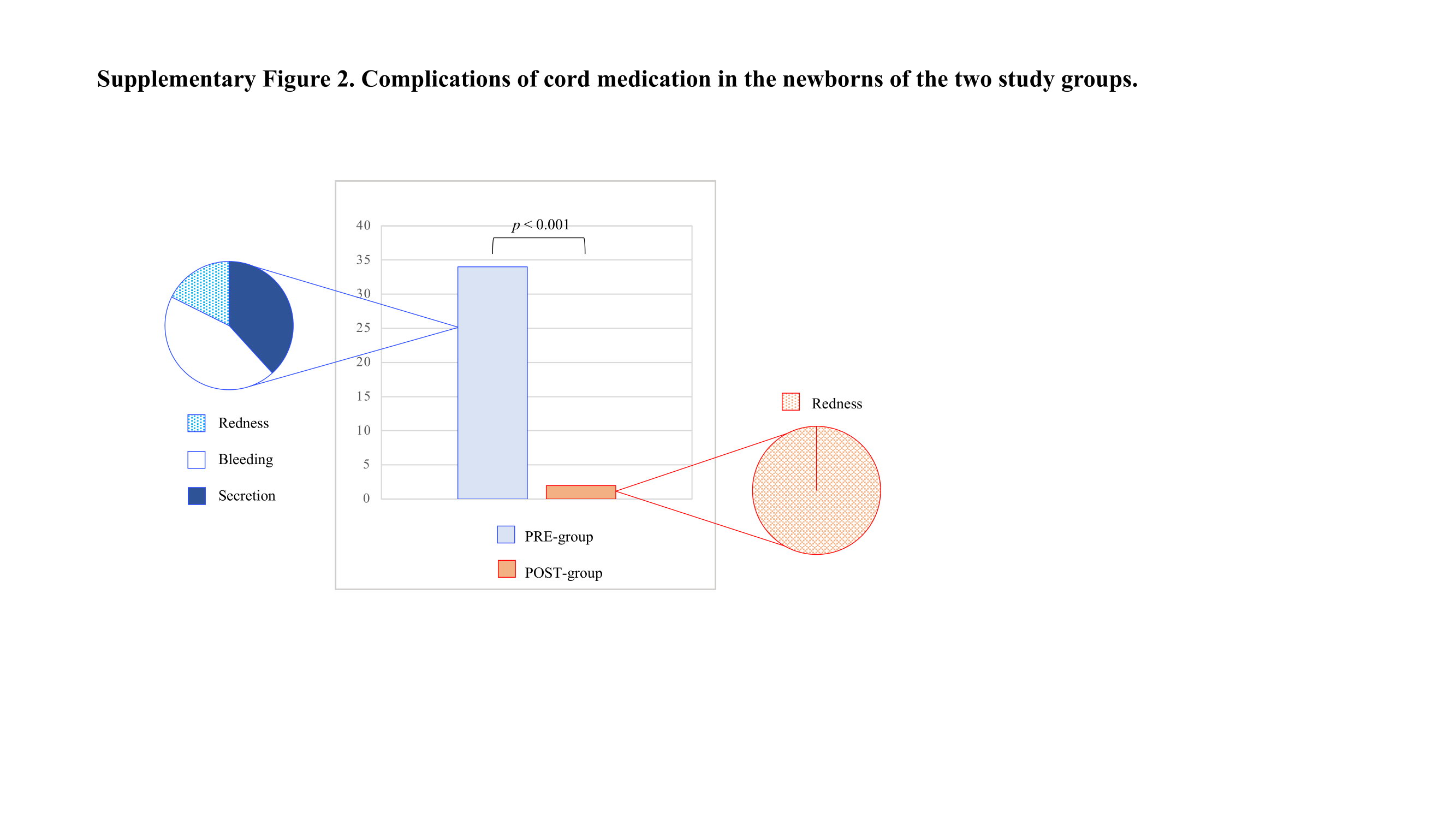

Supplement: Supplementary file 4 — High resolution (TIFF 130 kb) [file 431_2020_3889_MOESM2_ESM.tiff]

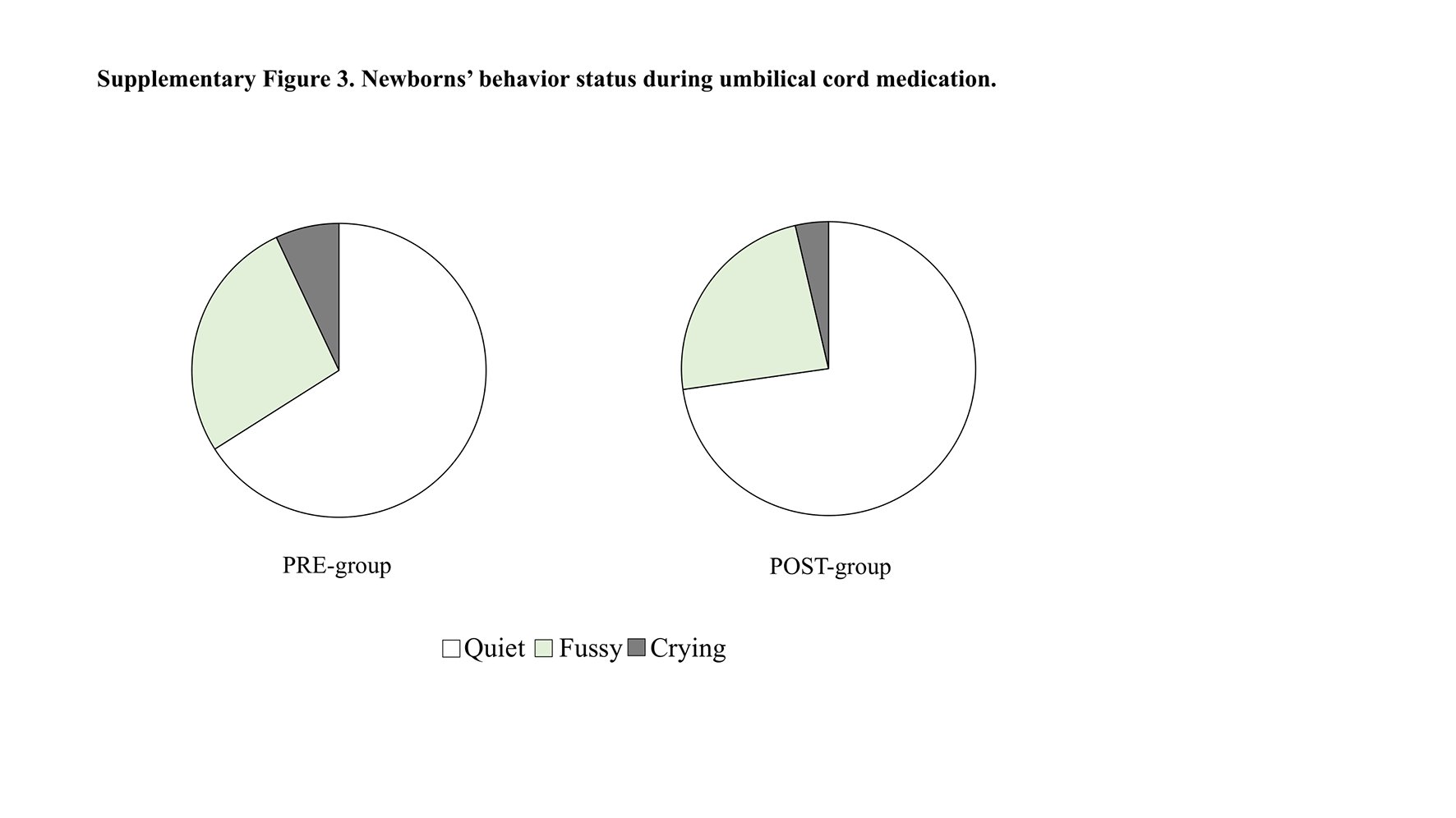

Supplement: Supplementary file 5 — (PNG 102 kb) [file 431_2020_3889_Fig5_ESM.png]

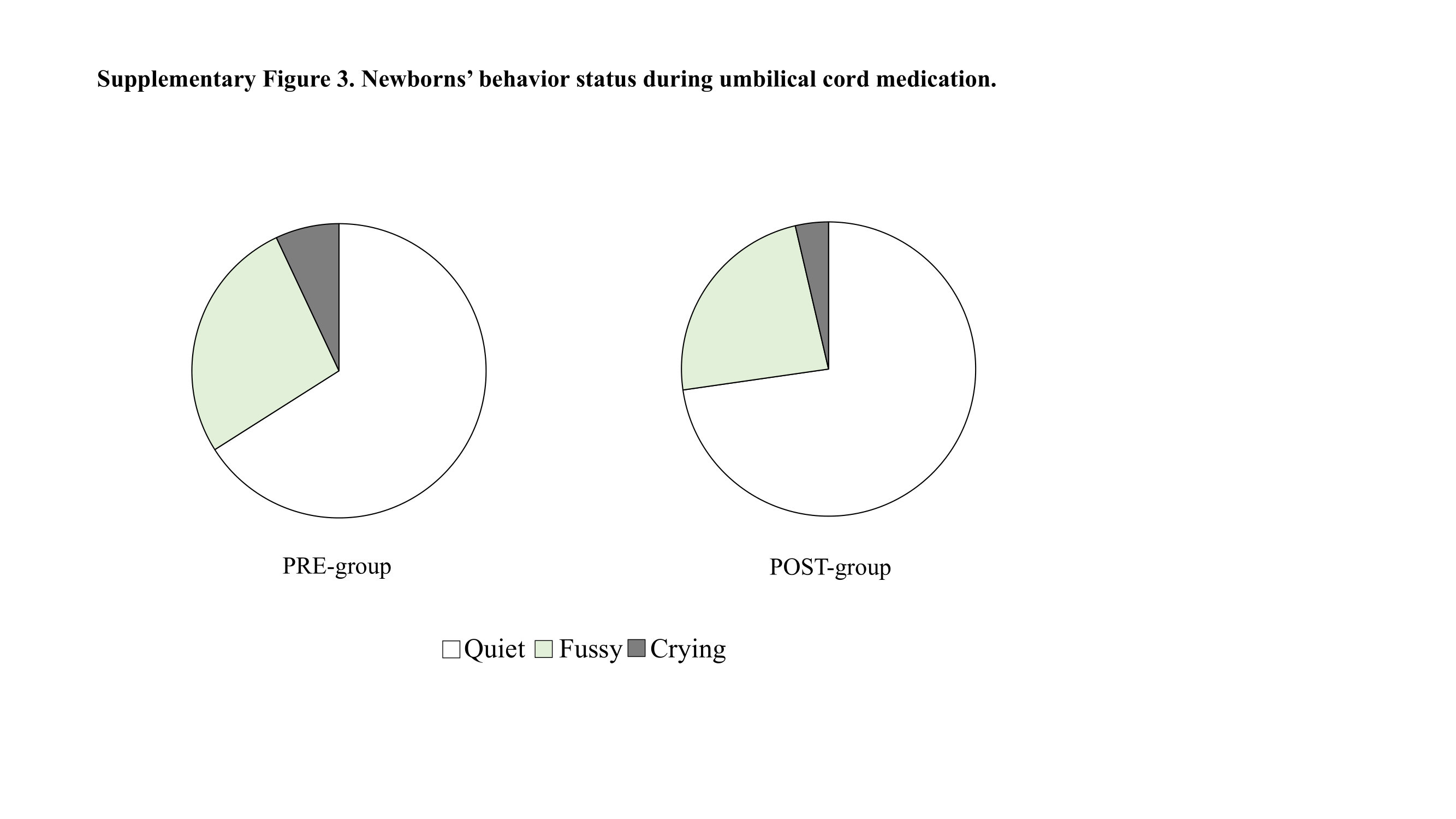

Supplement: Supplementary file 6 — High resolution (TIFF 124 kb) [file 431_2020_3889_MOESM3_ESM.tiff]
