# Supplementary material for: Umbilical cord medication in healthy full-term newborns: a before-after uncontrolled quality improvement study
Source: Eur J Pediatr. 2020 Dec 7;180(2):505–11. doi: 10.1007/s00431-020-03889-w (PMC7813727; doi:10.1007/s00431-020-03889-w)
Supplement: Supplementary file 7 — (DOCX 16 kb) [file 431_2020_3889_MOESM4_ESM.docx]

**SUPPLEMENTARY TABLE 1.** Regression analisys to evaluate the influence of covariates on the primary ouctome.

|  |  | *Std. Err.* | *B* | *p value* | *95% CI* |
| --- | --- | --- | --- | --- | --- |
| **Cord detachment timing** | *Birth Weight* | 0.000 | 0.000 | 0.149 | 0.999 to 1.000 |
| (chi-2= 114.206, *p* value < 0.001) | *5-min Apgar score* | 0.134 | 0.120 | 0.369 | 0.868 to 1.466 |
|  | *Spring season* | 0.206 | -0.657 | **0.001** | 0.346 to 0.777 |
|  | *Type of delivery* | 0.148 | 0.003 | 0.983 | 0.750 to 1.341 |
|  | *Primiparity* | 0.162 | 0.363 | **0.025** | 1.046 to 1.975 |
|  | *Male sex* | 0.158 | 0.149 | 0.346 | 0.852 to 1.581 |
|  | *Age of the mother* | 0.015 | 0.000 | 0.978 | 0.971 to 1.031 |
|  | *Cord difficulty level* | 0.038 | -0.003 | 0.937 | 0.925 to 1.075 |
|  | *Group assignment* | 0.197 | -1.422 | **< 0.001** | 0.164 to 0.355 |
